# Prediction of net energy values in expeller-pressed and solvent-extracted rapeseed meal for growing pigs

**DOI:** 10.5713/ajas.19.0962

**Published:** 2020-04-12

**Authors:** Zhongchao Li, Zhiqian Lyu, Hu Liu, Dewen Liu, Neil Jaworski, Yakui Li, Changhua Lai

**Affiliations:** 1 State Key Laboratory of Animal Nutrition, Ministry of Agriculture Feed Industry Centre, China Agricultural University, Beijing 100193, China; 2 Trouw Nutrition, Veerstraat 38, 5831 JN Boxmeer, The Netherlands

**Keywords:** Heat Production, Indirect Calorimetry, Net Energy, Prediction Equations, Rapeseed Meal, Pig

## Abstract

**Objective:**

The objective of this study was to determine net energy (NE) of expeller-press (EP-RSM) and solvent-extracted rapeseed meal (SE-RSM) and to establish equations for predicting the NE in rapeseed meal (RSM) fed to growing pigs.

**Methods:**

Thirty-six barrows (initial body weight [BW], 41.1±2.2 kg) were allotted into 6 diets comprising a corn-soybean meal basal diet and 5 diets containing 19.50% RSM added at the expense of corn and soybean meal. The experiment had 6 periods and 6 replicate pigs per diet. During each period, the pigs were individually housed in metabolism crates for 16 days which included 7 days for adaption to diets. On day 8, pigs were transferred to respiration chambers and fed their respective diet at 2,000 kJ metabolizable energy (ME)/kg BW^0.6^/d. Feces and urine were collected, and daily heat production was measured from day 9 to 13. On days 14 and 15, the pigs were fed at 890 kJ ME/kg BW^0.6^/d and fasted on day 16 for evaluation of fasting heat production (FHP).

**Results:**

The FHP of pigs averaged 790 kJ/kg BW^0.6^/d and was not affected by the diet composition. The NE values were 10.80 and 8.45 MJ/kg DM for EP-RSM and SE-RSM, respectively. The NE value was positively correlated with gross energy (GE), digestible energy (DE), ME, and ether extract (EE). The best fit equation for NE of RSM was NE (MJ/kg DM) = 1.14×DE (MJ/kg DM)+0.46×crude protein (% of DM)–25.24 (n = 8, R^2^ = 0.96, p<0.01). The equation NE (MJ/kg DM) = 0.22×EE (% of DM)–0.79×ash (% of DM)+14.36 (n = 8, R^2^ = 0.77, p = 0.018) may be utilized to quickly determine the NE in RSM when DE or ME values are unavailable.

**Conclusion:**

The NE values of EP-RSM and SE-RSM were 10.80 and 8.45 MJ/kg DM. The NE value of RSM can be well predicted based on energy content (GE, DE, and ME) and proximate analysis.

## INTRODUCTION

In the past few years, the price and demand of soybean meal, the most used protein ingredient for swine, have risen dramatically. Double-low rapeseed meal (RSM) also called canola meal in North American, Australia and Asian is a well-known alternative protein supplement for swine [1,2]. Expeller-pressed RSM (EP-RSM) and solvent-extraction RSM (SE-RSM) are two primary RSMs for livestock feed. Chemical composition and energy value in RSM are extremely variable [3]. Use of the net energy (NE) system in diet formulation allows for a more effective use of high fiber ingredients, such as RSM, wheat bran and oat bran [4,5]. Several research projects have been conducted to evaluate the NE value of RSM [6,7]. Because of the large variation in chemical composition of RSM, datasets of energy value based on many different RSM samples and sources are necessary. Furthermore, NE determination requires sophisticated equipment and is time-consuming and, therefore, impractical to determine NE value in every batch of RSM before diet formulation. Prediction equations based on proximate analysis and datasets of feed ingredient digestible energy (DE) and metabolizable energy (ME) values allow rapid estimation of feed ingredient NE values. However, to our knowledge, there is no equation for predicting the NE value of RSM [7,8].

Therefore, the objective of this study was to determine the NE value of EP-RSM and SE-RSM and, subsequently, to establish equations for predicting the NE in RSM based on proximate analysis and a database of DE and ME of 8 different RSM samples previously analyzed by our laboratory. We hypothesized that the prediction equations can be established and predict the NE of RSM well.

## MATERIALS AND METHODS

This experiment was reviewed by “Department of China Agricultural University Animal Care and Use Ethics Committee” (AW17129102-1, Beijing, China) which was approved by the Institutional Animal Care and Use Committee of China Agricultural University.

### Collection of rapeseed meal samples

A total of 12 RSM samples (6 EP-RSM and 6 SE-RSM, Supplementary Table S1) were obtained from commercial feed mills across China based on a range of ether extract (EE) of 0.87% to 11.27% in the hopes that the prediction equations could be applied to a wide range of RSM samples. Five samples were selected based on their chemical composition using cluster analysis (data not shown). Source 2 of EP-RSM and source 3 SE-RSM contained more than 30 μmol/g total glucosinolates. These 2 sources of RSM were not processed from double low rapeseed, the other samples were double low RSM.

### Animals, diets and experimental design

The experiment was conducted at the FengNing Swine Research Unit of China Agricultural University (Hebei Province, China). Thirty-six growing barrows (Duroc×Large White× Landrace, initial body weight [BW] = 41.1±2.2 kg) were allotted into 1 of 6 dietary treatments in a completely randomized design with 6 pigs per diet. Diets included a corn-soybean meal-based basal diet and 5 test diets containing 19.50% RSM added at the expense of corn and soybean meal. The experiment lasted for 6 periods due to availability of 6 open-circuit respiration chambers previously described by Zhang et al [9].

During each period, pigs were individually housed in metabolism crates for 16 d, which included 7 d to adapt to the feed, metabolism crate, and environmental conditions. On d 8, the pigs were transferred to the open-circuit respiration chambers for measurement of daily O_2_ consumption and CO_2_ and CH_4_ production. During this time, pigs were fed at 2,000 kJ ME/kg BW^0.6^/d. The feeding level based on a preliminary experiment was used to avoid stress and diarrhea. Total feces and urine were collected and daily heat production (HP) was measured from d 9 to d 13. On d 14 and 15, pigs were fed at their maintenance requirement level (ME_m_ = 890 kJ ME/kg BW^0.6^/d) to adapt from the fed to the fasted state [9]. The HP was also measured at this low feed level, but the results are not included in the present paper. On the last day of each period (d 16), pigs were fasted and fasting heat production (FHP) corresponded to the HP measured during the last 8 hours of d 16 from 22:30 (d 16) to 06: 30 (d 17). The FHP period started 31 h after the last meal and animals were kept in the dark during collection period to minimize physical activity.

### Sample collection

During d 9 to 13, feed refusals and spillage were collected twice daily, dried and weighed. Feces were collected twice daily at 08:30 and 15:30 h when the chamber door was opened and immediately stored at −20°C. Urine was collected each morning at 08:30 h for each pig from plastic buckets containing 50 mL of 6 *N* HCl and filtered through cotton gauze. Total urine volume produced by each pig was measured and 5% of the daily urine excretion was stored at −20°C. At the end of urine collection, samples were thawed, and thoroughly mixed, and a sub-sample was saved for analysis. Urine was collected separately during the 24 h fasting state to calculate urinary N losses for the calculation of FHP.

At the end of the experiment, fecal samples were thawed, mixed, weighed, and sub-samples were oven-dried for 72 h at 65°C. The feed and fecal samples were ground through a 1-mm screen prior to chemical analysis.

### Chemical analysis

Ingredients, diets and feces were analyzed for dry matter (DM; method 930.15, [10]) and organic matter (OM) was calculated as DM minus ash content. Crude protein (CP, method 984.13 [10]), ash (method 942.05 [10]), and EE [11] were analyzed in ingredients, diets, and feces. The neutral detergent fiber (NDF) and acid detergent fiber (ADF) were determined using filter bags and fiber analyzer equipment (Fiber Analyzer, Ankom Technology, Macedon, NY, USA) following a modification of the procedure of Van Soest et al [12]. Total glucosinolate concentration was analyzed in ingredients according to Daun and McGregor [13]. The gross energy (GE) in the five RSMs, diets, feces, and urine samples were analyzed using an isoperibol calorimeter (Parr 6300 Calorimeter, Moline, IL, USA) with benzoic acid as a standard. The five RSMs and diets were also analyzed for total starch by the glucoamylase procedure (Method 948.02 [10]).

### Calculations

All calculations were similar to those reported by Li et al [7]. In brief, the DM intake from d 9 to 13 in each period was calculated as the product of feed intake and DM content of diets. The GE intake was calculated as the product of the GE content of the diet and the actual feed DM intake over the 5-d collection period from d 9 to 13. The energy lost in feces, urine, and methane was measured for each animal on a given diet. The ME included energy lost as urine and methane. Energy lost as methane was calculated using the 39.54 kJ/L conversion factor [14].

Total heat production (THP) was then calculated for each day from gas exchanges and urinary loss of N according to Brouwer [14] using Eq. (1):

(1) $$\text{THP }(\text{kJ})=16.18\times {\text{O}}_{2}\;(\text{L})+5.02\times {\text{CO}}_{2}\;(\text{L})-2.17\times {\text{CH}}_{4}\;(\text{L})-5.99\times \text{urinary N }(\text{g})$$

Retention of energy (RE) was calculated according to Eq. (2):

(2) RE (MJ/kg DM)=[ME intake (MJ/d)-THP (MJ/d)]/DM intake (kg/d)

Retention of energy as protein (RE_P_) was calculated as N retention (g)×6.25×23.86 (kJ/g). Retention of energy as lipid (RE_L_) was calculated as the difference between RE and RE_P_.

The FHP was calculated using the equation used for THP with gas concentrations and air flow obtained from the last 8-hours on d 16 (i.e. from 22:30 to 06:30 h; [9]). To base FHP using the same time span as used for THP, the 8-h FHP was extrapolated to a 24-h period. The NE of each diet was calculated according to Noblet et al [15] using Eq. [3]:

(3) NE (kJ/kg DM)=[RE (kJ/d)+FHP (kJ/d)]/DM intake (kg/d)

The DE, ME, and NE of the basal diet was divided by 0.975 (the DM ratio of corn plus soybean meal in the basal diet) to calculate the DE, ME, and NE of the corn and soybean meal mixture. The difference method [16] was used to calculate the average GE, DE, ME, and NE contributions of each RSM from the mean GE, DE, ME, and NE contents of each diet and assuming that the average GE, DE, ME, and NE of the corn and soybean mixture obtained for the basal diet was applicable to the other diets. The DE/GE, ME/DE, and NE/ME ratios could then be calculated for each RSM from these calculated GE, DE, ME, and NE values and used to estimate the final DE, ME, and NE values as the product of measured GE and DE/GE for DE, measured GE and DE/GE and ME/DE for ME and measured GE and DE/GE, ME/DE, and NE/ME for NE. All calculations were done on a DM basis. The respiratory quotient was calculated as the ratio between CO_2_ production and O_2_ consumption. The apparent total tract digestibility (ATTD) of nutrients in diets was calculated according to the methods of Noblet et al [15].

### Statistical analysis

Data were subjected to analysis of variance using the PROC MIXED procedure of SAS (SAS Inst. Inc., Carry, NC, USA) with diet as the fixed effect and period and chamber as random effects. The differences were considered significant if p<0.05. The relationship between energy content (GE, DE, ME, and NE) and chemical composition of 8 RSM samples was determined using PROC CORR of SAS. Prediction equations for NE of 8 RSM samples were developed using PROC REG of SAS. The R^2^, root mean square error (RMSE) and Akaike’s information criterion (AIC) were used as the selection criterion for the best fit equations. The equations with the greatest R^2^ and the least RMSE were proposed to indicate the best fit. Data of five RSM samples reported in the current study and that of two samples (Supplementary Table S2) were from Li et al [7], and one sample was from Liu et al [6].

## RESULTS

### Chemical composition of rapeseed meal

The chemical composition of RSM samples and diets are shown in Table 1 and 2, respectively. The EP-RSM contained 8.9% EE and SE-RSM contained 2.0% EE. The averaged CP concentration in the EP-RSM was 38.7% and in the SE-RSM it was 40.9%. Source 1 EP-RSM contained the highest EE among the 5 RSMs (11.3%). The NDF content in the EP-RSM ranged from 32.3% to 41.0%, which is similar to the range of SE-RSM (30.8% to 39.8%). The composition of the 6 test diets was consistent with differences in ingredient composition with the EP-RSM diets containing 4.1% EE and the SE-RSM diets containing 2.7% EE.

### Nutrients digestibility and nitrogen balance for diets

The ATTD of NDF, ADF, EE, and GE in the EP-RSM diets were greater (p<0.05) than in the SE-RSM diets (Table 3). The nitrogen intake and output from feces by pigs fed SE-RSM diets were greater (p<0.01) than EP-RSM diets, while the urinary nitrogen output was similar, which led to greater (p<0.05) nitrogen retention in pigs fed SE-RSM diets than pigs fed EP-RSM diets (25.3 vs 21.7 g/d).

### Energy balance and energy value for experimental diets

Despite no difference in ME intake (Table 4), THP, and REP, pigs fed the diets containing EP-RSM had greater (p<0.05) RE_L_ compared with pigs fed diets containing SE-RSM (419 vs 326 kJ/kg BW^0.6^/d). The respiratory quotient was the greatest (p<0.01) in the basal diet due to the greater dietary starch content compared with the test diets. The FHP averaged 790 (range, 752 to 828) kJ/kg BW^0.6^/d and was not affected by diet composition.

The NE to ME ratio averaged 77.3 and 75.6 MJ/kg DM for EP-RSM and SE-RSM diets, respectively, and was not affected by diets. The EP-RSM diets had greater (p<0.01) DE, ME, and NE compared with SE-RSM diets (15.94 vs 15.36 MJ/kg DM for DE, 15.18 vs 14.70 MJ/kg DM for ME, 11.74 vs 11.23 MJ/kg DM for NE).

### Nutrient digestibility of nutrients and energy contents for ingredients

Compared with source 1, 2 and 4, source 3 and 5 of SE-RSM had lower (p<0.05) ATTD of CP, OM, and GE in connection with their higher NDF, which also led to a lower (p<0.05) NE for these 2 SE-RSM samples (Table 5). The average nutrient digestibility in pigs fed EP-RSM were greater (p<0.05) than pigs fed SE-RSM. The ME to DE ratio (90.2% vs 91.2%) was not different between EP-RSM and SE-RSM, while a greater (p<0.05) NE to ME ratio was calculated for EP-RSM (76.2% vs 71.1%) compared with SE-RSM. The average DE, ME, and NE values for EP-RSM were greater (p<0.05) than that in SE-RSM. The average energy values for EP-RSM and SE-RSM were 15.72 and 13.05 MJ/kg DM for DE, 14.17 and 11.90 MJ/kg DM for ME, and 10.80 and 8.45 MJ/kg DM for NE, respectively.

### Correlation analysis and net energy prediction equations for eight rapeseed meal samples

The EE content had a positive correlation with GE (r = 0.98, p<0.01), DE (r = 0.89, p<0.01), ME (r = 0.87, p<0.01), and NE (r = 0.76, p<0.05), while the EE content was negatively correlated with CP content (r = −0.83, p<0.01; Table 6). The NDF content was positively correlated with ADF content (r = 0.93, p<0.01).

The stepwise regression equations for NE in RSM samples are presented in Table 7. Energy values (GE, DE, ME, and NE) had a high correlation among themselves (p<0.01) and, therefore, the regression equations for NE in the current experiment were developed based on only chemical composition, or chemical composition and a single energy system (GE, DE, or ME). When the stepwise regression equations were only based on chemical composition, EE was the first predictor for NE of RSM, but the accuracy of the equations was improved if ash was included in the prediction equation (NE = 0.22×EE−0.79×ash+14.36, R^2^ = 0.77, p = 0.018). The accuracy of equations based on GE was improved when the equation included EE (NE = 4.62×GE–0.67×EE–80.43, R^2^ = 0.82, p = 0.014). The CP improved the accuracy of equation based on DE (NE = 1.14×DE+0.46×CP–25.24, R^2^ = 0.96, p<0.01). The ME was the single predictor to predict the NE value of RSM when stepwise regression equation was based on ME (NE = 0.85×ME–1.48, R^2^ = 0.88, p<0.01).

## DISCUSSION

### Chemical composition of rapeseed meal

The source of raw rapeseed and differences in processing conditions may result in RSM of variable chemical composition [3]. Source 1 EP-RSM contained 11.27% EE while source 2 contained 6.55% EE which indicates that the expeller conditions were more extreme for source 2 compared with source 1. In fact, source 1 was not heat treated and source 2 was heated at temperature from 110°C to 120°C. Similarly, differences in NDF content between source 1 and 2 indicate that the rapeseed processed to produce source 1 EP-RSM contained more hulls than source 2. The solvent extraction procedure was more efficient for oil extraction than expeller pressed procedure [17], which led to a relatively low residual oil content in the SE-RSM.

The average EE content in the EP-RSM and SE-RSM observed in the current study is lower than the average reported by the NRC [18] (8.9% vs 10.7% for EP-RSM, 2.0% vs 3.5% for SE-RSM, DM basis). These differences likely indicate advancement in oil extraction processes used for the oil extraction from rapeseed. The CP content in RSM was similar to the value reported by NRC [18]. The content of NDF in the RSM used in the current experiment is in the range of our previous studies reported by Li et al [2,3] using RSM also produced in China. However, the NDF content in the current RSM samples and those used previously by our group [2,3] were at or above the upper range of that reported in NRC [18]. Differences in variety and analytical methods or result of Maillard reactions during desolventization and toasting may explain the discrepancy of NDF [19].

### Nutrients digestibility and nitrogen balance for diets

The digestibility of EE was greater in EP-RSM diets compared with SE-RSM diets and this may be attributed to the greater EE content in EP-RSM. Previous studies showed that the ATTD of EE increased with increasing dietary fat [20,21], indicating that the endogenous fat has a greater effect on the ATTD of fat at low dietary levels than at higher levels [20,21]. The digestibility of NDF, ADF, and GE in EP-RSM is accentuated by the high digestible oil fraction, which may be the reason that the greater EE contents have decreased passage rate of digesta resulting in increased ATTD of NDF [22]. As in previous experiments, the digestibility of DM and OM were less in the 5 RSM diets compared with the basal diet and this may be due to the greater NDF content in RSM [23].

### Energy balance and energy value for experimental diets

The THP was not affected by diet composition and this result agreed with that reported by Lyu et al [5]. Pigs fed EP-RSM retained more fat than pigs fed SE-RSM. It can be explained that EP-RSM contained greater EE content than SE-RSM. In the current experiment, the FHP was estimated as the nocturnal HP after a period of feed deprivation of 31 h to minimize physical activity. The FHP value was within the range of values for FHP estimated in our previous work conducted in the same facility [5,7], and the FHP estimated in the current experiment is slightly greater than the mean FHP value (750 kJ/kg BW^0.6^/d) reported by Noblet et al [15]. However, the estimation of NE for maintenance (or FHP) influences directly the absolute NE value of a feedstuff [9]. Many factors can contribute to differences in FHP between Noblet and current study, which included previous feeding levels [24], duration of the fasting period [9], methodology [6], and physical activity [25]. Therefore, caution should be used when determining the NE value of feedstuffs using a FHP value from literature. Corresponding to the THP, it is highly preferable to calculate the NE value of feedstuffs from the individual FHP for the same pig to attenuate the effect of the variation for measurement of FHP. In agreement with other literature [26,27], the FHP was not affected by diet composition.

Efficiency of utilization of ME for NE depends on the chemical composition of a feed ingredient [28]. This observation agrees with the conclusion that lipids have a greater efficiency of utilization of ME [25]. This apparent discrepancy is likely related to the greater NE:ME ratio determined in this study compared to NRC [18] which may be due to higher FHP measured in the current study. These results also confirm that caution should be used when comparing measured NE values with predicted values from literature equations.

The average DE and ME content of EP-RSM and SE-RSM are less than the values reported by NRC [18], respectively and this may be caused by a lower EE and higher NDF and ADF content in the EP-RSM used in the present experiment. The NE values of EP-RSM and SE-RSM predicted by equations in the current experiment are higher than values predicted by equations from Noblet et al [15] and NRC [18]. This is because FHP in the current study was measured using the fasting method, while FHP was adopted by Noblet et al [15] using the regression method. That caused the FHP used in the current study to be greater than that (750 kJ/kg BW^0.6^/d) reported by Noblet et al [15].

### Correlation analysis and net energy prediction equations

Noblet et al [15] generated a series of prediction equations for NE based on complete diets, however, caution is essential when applying predictions to individual ingredients [18]. The current study is the first to develop prediction equations for NE of RSM based on chemical composition and GE, DE, or ME. In our previous work [6,7], the NE for 3 RSM samples have been determined using the same procedure. Data on energy value of the 5 samples used in the current study were combined with energy value of 3 additional rapeseed samples previously determined by this research group. Therefore, in the current experiment, total 8 RSM samples (3 EP-RSM and 5 SE-RSM) were used to analyze the correlation coefficients and to develop the NE prediction equations.

The strong negative correlation between EE and CP confirmed the dilution effect of residual oil in RSM; as more oil is removed from rapeseed, the CP concentration is increased [7]. The observation that EE was positively correlated with energy value (GE, DE, ME, or NE) agrees with previous data [7,29]. These reports also indicated that energy values (GE, DE, ME, or NE) had a high correlation among themselves, which also was observed in the current experiment.

Regression equations for NE were first developed based on proximate analysis of chemical composition. The EE predominantly predicted the NE value of RSM and this was expected because fat contains more energy compared with carbohydrates and CP. Prediction equation 4 was the best fit to predict the NE value of RSM when data of energy value was not included and represents a suitable equation for rapid prediction of NE in RSM when specific energy values are not available. The accuracy of the equation was improved when GE was included in the prediction. When data of DE or ME for RSM are available, equation 1 and 2 predicted the NE value of RSM more accurately. Considering the statistical criterion of R^2^, RMSE, and AIC, equation 1 (based on DE) may be the best fit to predict the NE value of RSM.

## CONCLUSION

The NE value was 10.80 and 8.45 MJ/kg DM for EP-RSM and SE-RSM, respectively. The NE in RSM was positively correlated with GE, DE, ME, and EE. The best fit equation for NE of RSM was NE (MJ/kg DM) = 1.14×DE (% of DM) +0.46×CP (% of DM)–25.24 (n = 8, R^2^ = 0.96, p<0.01). However, the equation NE (MJ/kg DM) = 0.22×EE (% of DM)– 0.79×ash (% of DM)+14.36 (n = 8, R^2^ = 0.77, p<0.05) may be a suitable alternative for rapid determination of NE in RSM when DE or ME are not available.

## Figures and Tables

**Table 1:** Analyzed nutrient composition of rapeseed meal used in the experiment (DM basis)1)

Item	EP-RSM		SE-RSM		
	
	1	2	3	4	5
Dry matter (%)	93.53	91.65	91.48	89.90	90.82
Gross energy (MJ/kg)	21.35	20.56	19.47	19.60	19.37
Crude protein (%)	37.70	39.75	39.92	41.03	41.83
Ether extract (%)	11.27	6.55	2.58	1.71	1.68
Starch (%)	2.95	4.43	3.02	3.12	2.26
NDF (%)	41.00	32.34	37.27	30.84	39.83
ADF (%)	24.18	21.75	21.50	20.04	24.79
Crude fiber (%)	19.18	15.04	13.58	14.23	17.86
Ash (%)	7.49	6.94	8.24	7.22	9.14
Total glucosinolates (μmol/g)	11.27	39.42	48.70	9.12	8.31

DM, dry matter; EP-RSM, expeller-pressed rapeseed meal; SE-RSM, solvent-extracted rapeseed meal; NDF, neutral detergent fiber; ADF, acid detergent fiber.

1) All samples were analyzed in duplicate.

**Table 2:** Ingredient and analyzed chemical composition of experimental diets1)

Items	Basal diet	EP-RSM		SE-RSM		
	
		1	2	3	4	5
Ingredients (%)						
Corn	72.50	58.00	58.00	58.00	58.00	58.00
Soybean meal	25.00	20.00	20.00	20.00	20.00	20.00
Rapeseed meal	0.00	19.50	19.50	19.50	19.50	19.50
Dicalcium phosphate	0.90	0.90	0.90	0.90	0.90	0.90
Limestone	0.75	0.75	0.75	0.75	0.75	0.75
Salt	0.35	0.35	0.35	0.35	0.35	0.35
Vitamin and mineral premix2)	0.50	0.50	0.50	0.50	0.50	0.50
Total	100.00	100.00	100.00	100.00	100.00	100.00
Analyzed composition (%), as-fed						
Dry matter	88.17	89.06	88.71	88.81	88.69	88.69
Crude protein	16.62	20.12	20.69	20.68	20.78	21.07
Ether extract	2.42	4.46	3.78	2.70	2.69	2.61
Starch	46.26	39.47	39.06	38.79	40.04	38.89
Neutral detergent fiber	11.02	15.95	13.92	13.76	13.93	14.78
Acid detergent fiber	3.06	6.79	6.08	5.98	6.13	6.84
Ash	4.08	5.09	4.84	5.09	4.95	5.36
Calculated composition (%)						
SID lysine	0.76	0.95	0.95	0.94	0.94	0.96
SID methionine	0.20	0.27	0.27	0.27	0.27	0.27
SID threonine	0.57	0.67	0.69	0.68	0.69	0.72
SID threonine	0.15	0.20	0.20	0.20	0.20	0.21
SID valine	0.65	0.79	0.79	0.80	0.80	0.80
SID leucine	1.35	1.47	1.48	1.48	1.48	1.48
SID isoleucine	0.56	0.66	0.65	0.66	0.66	0.66
SID histidine	0.40	0.50	0.50	0.50	0.50	0.50
SID tyrosine	0.47	0.51	0.51	0.50	0.50	0.50
SID cysteine	0.21	0.29	0.29	0.29	0.29	0.29

EP-RSM, expelled press rapeseed meal; SE-RSM, solvent extracted rapeseed meal; SID, standardized ileal digestibility.

1) All samples were analyzed in duplicate.

2) Vitamin-mineral premix supplied the following per kg of diet: vitamin A, 5,512 IU; vitamin D_3_, 2,200 IU; vitamin E, 30 IU; vitamin K_3_, 2.2 mg; vitamin B_12_, 27.6 μg; riboflavin, 4 mg; pantothenic acid, 14 mg; niacin, 30 mg; choline chloride, 400 mg; folic acid, 0.7 mg; thiamine, 1.5 mg; pyridoxine, 3 mg; biotin, 44 μg; Mn (MnO), 40 mg; Fe (FeSO_4_·H_2_O), 75 mg; Zn (ZnO), 75 mg; Cu (CuSO_4_·5H_2_O), 100 mg; I (KI), 0.3 mg; Se (Na_2_SeO_3_), 0.3 mg.

**Table 3:** Effect of diet characteristics on energy and nitrogen balances of growing pigs1)

Items	Basal diet	EP-RSM		SE-RSM			SEM	p-value2)	Mean values		SEM	p-value3)
		
		1	2	3	4	5			LSM-EP	LSM-SE		
Body weight (kg)	47.82	45.93	47.53	46.53	48.07	47.48	1.55	0.513	46.73	47.36	1.21	0.574
Dry matter intake (kg/d)	1.30	1.30	1.30	1.33	1.35	1.35	0.02	0.121	1.30	1.34	0.02	0.002
ATTD (%)												
Dry matter	89.6^a^	85.7^bc^	86.2^b^	84.7^bc^	85.8^bc^	83.4^c^	0.7	0.002	85.9	84.6	0.8	0.058
Crude protein	87.2^a^	85.5^ab^	85.9^a^	84.1^ab^	85.4^ab^	81.7^b^	1.0	0.004	85.7	83.7	1.3	0.052
Organic matter	91.0^a^	87.6^bc^	88.1^b^	86.8^bc^	87.6^bc^	85.4^c^	0.6	0.008	87.9	86.6	0.4	0.050
Neutral detergent fiber	57.3^a^	57.0^a^	51.3^ab^	47.6^ab^	49.9^ab^	43.9^b^	2.7	0.012	54.2	47.1	2.3	0.018
Acid detergent fiber	52.4^a^	48.4^ab^	43.2^ab^	37.1^b^	45.2^ab^	34.9^b^	3.3	0.092	45.8	39.1	2.8	0.044
Ether extract	47.9^c^	67.1^a^	67.2^a^	57.0^b^	49.5^bc^	50.7^bc^	2.4	0.003	67.2	52.4	1.7	0.004
Gross energy	88.7^a^	85.3^bc^	85.9^ab^	84.4^bc^	85.2^bc^	82.9^c^	0.7	0.008	85.6	84.2	0.9	0.043
Nitrogen balance (g/d)												
Intake	39.3^d^	46.9^c^	48.4^bc^	49.3^ab^	50.6^ab^	51.3^a^	0.9	0.006	47.6	50.4	1.1	0.004
Fecal output	5.0^c^	6.8^bc^	6.8^bc^	7.8^ab^	7.4^b^	9.4^a^	0.5	0.003	6.8	8.2	0.7	0.012
Urinary output	13.5	22	16.3	17.2	17.6	16.0	2.0	0.074	19.1	16.9	2.3	0.185
Retention	20.7^ab^	18.1^b^	25.3^a^	24.4^ab^	25.6^a^	25.9^a^	1.9	0.010	21.7	25.3	2.3	0.050

EP-RSM, expelled press rapeseed meal; SE-RSM, solvent extracted rapeseed meal; SEM, standard error of the mean; LSM-EP is the mean of expelled press rapeseed meal; LSM-SE is the mean of solvent-extracted rapeseed meal; ATTD, apparent total tract digestibility.

1) Data are means of six observations.

2) This p-value is the comparison of five sources of rapeseed meal.

3) This p-value is the comparison of the mean of expelled press rapeseed meal and solvent-extracted rapeseed meal.

a–d Means in the same row with differing superscripts differ (p<0.05).

**Table 4:** Effect of diet characteristics on energy balance of growing pigs1)

Items	Basal diet	EP-RSM		SE-RSM			SEM	p-value2)	Mean values		SEM	p-value3)
		
		1	2	3	4	5			LSM-EP	LSM-SE		
ME intake	1,989	1,979	1,951	1,949	1,963	1,934	26	0.632	1,965	1,949	19	0.478
Total heat production	1,214	1,207	1,235	1,255	1,273	1,222	31	0.528	1,221	1,250	26	0.283
Fasting heat production	775	752	816	788	828	780	26	0.283	784	798	33	0.571
REP4)	304	277	373	363	374	381	29	0.065	325	373	20	0.057
REL4)	471^ab^	495^a^	344^bc^	331^bc^	316^c^	331^bc^	35	0.001	419	326	25	0.010
Total RE	775	772	716	694	691	712	34	0.092	744	699	31	0.083
Respiratory quotient												
Fed state	1.10^a^	1.07^b^	1.06^b^	1.06^b^	1.06^b^	1.07^b^	0.01	0.001	1.07	1.06	0.01	0.265
Fasted state	0.81	0.81	0.81	0.81	0.80	0.81	0.01	0.643	0.81	0.81	0.01	0.492
Energy utilization (%)												
Urinary energy % of DE	2.5^b^	4.3^a^	3.6^ab^	3.7^ab^	3.9^ab^	3.4^ab^	0.4	0.032	3.9	3.7	0.6	0.393
ME/DE	96.6	95.0	95.6	95.8	95.3	95.9	0.4	0.104	95.3	95.7	0.3	0.222
NE/ME	77.5	76.6	78.0	75.6	77.1	76.7	0.8	0.151	77.3	76.5	0.6	0.143
Energy values (MJ/kg DM)												
DE	16.08^a^	15.94^ab^	15.95^ab^	15.38^c^	15.55^bc^	15.15^c^	0.13	0.002	15.94	15.36	0.16	0.003
ME	15.54^a^	15.13abc	15.24^ab^	14.73^cd^	14.83^cd^	14.53^d^	0.13	0.002	15.18	14.70	0.19	0.004
NE	12.05^a^	11.59abc	11.89^ab^	11.13^c^	11.43^bc^	11.14^c^	0.15	0.003	11.74	11.23	0.16	0.008

EP-RSM, expelled press rapeseed meal; SE-RSM, solvent extracted rapeseed meal; SEM, standard error of the mean; LSM-EP, the mean of expelled press rapeseed meal; LSM-SE, the mean of solvent-extracted rapeseed meal; ME, metabolizable energy; RE_P_, retention of energy as protein; RE_L_, retention of energy as lipid; RE, Retention of energy; DE, Digestible energy; NE, Net energy, DM, dry matter.

1) Data are means of six observations.

2) This p-value is the comparison of five sources of rapeseed meal.

3) This p-value is the comparison of the mean of expelled press rapeseed meal and solvent-extracted rapeseed meal.

4) RE_P_ (kJ/kg BW^0.6^/d) = [N intake (g) – N in feces (g) – N in urine (g)]×6.25×23.86 (kJ/g)/BW^0.6^; REL (kJ/kg BW^0.6^/d) = [RE (kJ) – energy retention as protein (kJ)]/BW^0.6^.

a–d Means in the same row with differing superscripts differ (p<0.05).

**Table 5:** Energy utilization and energy value of the five ingredients1)

Items	EP-RSM		SE-RSM			SEM	p-value2)	Mean values		SEM	p-value3)
		
	1	2	3	4	5			LSM-EP	LSM-SE		
ATTD (%)											
Crude protein	82.3^a^	83.4^a^	78.6^b^	82.1^a^	72.2^c^	2.8	0.003	82.9	77.6	1.2	0.003
Organic matter	73.9^b^	76.3^a^	69.5^c^	73.6^b^	62.1^d^	1.6	0.001	75.1	68.4	2.3	0.002
Neutral detergent fiber	56.7^a^	41.0^bc^	30.4^c^	46.5^b^	28.8^c^	3.2	0.021	48.9	35.2	2.6	0.021
Acid detergent fiber	46.2^a^	36.9^b^	26.5^c^	40.4^ab^	25.1^c^	2.6	0.043	41.6	30.7	3.1	0.033
Ether extract	81.9^b^	87.5^a^	74.6^c^	61.6^d^	63.7^cd^	3.3	0.010	84.7	66.6	2.4	0.024
Gross energy	73.9^b^	76.1^a^	68.2^c^	71.8^b^	60.9^d^	1.4	0.004	75.0	67.0	1.0	0.001
Energy utilization (%)											
ME/DE	88.97^c^	91.38^ab^	91.88^a^	89.54^bc^	92.18^a^	0.9	0.023	90.18	91.2	0.7	0.343
NE/ME	72.21^c^	80.13^a^	65.34^d^	75.14^b^	72.68^c^	0.6	0.041	76.17	71.1	0.6	0.042
Energy values (MJ/kg DM)											
DE	15.78^a^	15.65^a^	13.28^bc^	14.07^b^	11.81^d^	0.12	0.001	15.72	13.05	0.5	0.008
ME	14.04^a^	14.30^a^	12.21^b^	12.60^b^	10.88^c^	0.21	0.004	14.17	11.90	0.4	0.006
NE	10.14^b^	11.46^a^	7.98^c^	9.47^b^	7.91^c^	0.20	0.022	10.80	8.45	0.8	0.021

EP-RSM, expelled press rapeseed meal; SE-RSM, solvent extracted rapeseed meal; SEM, standard error of the mean; LSM-EP, the mean of expelled press rapeseed meal; LSM-SE, the mean of solvent-extracted rapeseed meal; ATTD, apparent total tract digestibility; ME, metabolizable energy; DE, digestible energy; NE, net energy.

1) There were 6 pigs per treatment.

2) This p-value is the comparison of five sources of rapeseed meal.

3) This p-value is the comparison of the mean of expelled press rapeseed meal and solvent-extracted rapeseed meal.

a–d Within a row means followed by the same letters are not different at p<0.05.

**Table 6:** Correlation coefficients between chemical composition and energy values of the eight rapeseed meal samples1)

Items	CP	EE	Starch	NDF	ADF	Ash	GE	DE	ME	NE
CP	1.00	-	-	-	-	-	-	-	-	-
EE	−0.83**	1.00	-	-	-	-	-	-	-	-
Starch	−0.33	−0.01	1.00	-	-	-	-	-	-	-
NDF	−0.32	0.53	−0.69	1.00	-	-	-	-	-	-
ADF	−0.13	0.50	−0.75*	0.93**	1.00	-	-	-	-	-
Ash	0.30	−0.34	−0.54	0.55	0.49	1.00	-	-	-	-
GE	−0.78*	0.98**	0.06	0.40	0.40	−0.49	1.00	-	-	-
DE	−0.75*	0.89**	0.17	0.19	0.19	−0.65	0.93**	1.00	-	-
ME	−0.67	0.87**	0.09	0.25	0.27	−0.61	0.91**	0.98**	1.00	-
NE	−0.49	0.76*	0.13	0.08	0.21	−0.67	0.84**	0.93**	0.94**	1.00

CP, crude protein; EE, ether extract; NDF, neutral dietary fiber; ADF, acid dietary fiber; GE, gross energy; DE, digestible energy; ME, metabolizable energy; NE, net energy.

1) Data of five rapeseed meal samples were reported in the current study and that of two samples were from Li et al [29] and one sample were from Liu et al [6].

* p<0.05;

** p<0.01.

**Table 7:** Stepwise regression equations to estimate net energy in rapeseed meal1)

Number	Equations for rapeseed meal NE (n = 8)2)	Statistics			

		R^2^	AIC	RMSE	p-value
1	NE = 1.14×DE+0.46×CP–25.24	0.96	−14.14	0.36	0.005
2	NE = 0.85×ME–1.48	0.88	−7.69	0.56	0.007
3	NE = 4.62×GE–0.67×EE–80.43	0.82	−2.29	0.75	0.014
4	NE = 0.22×EE–0.79×ash+14.36	0.77	−0.53	0.84	0.018

NE, net energy; AIC, Akaike’s information criterion; RMSE, root mean square error; DE, digestible energy; CP, crude protein; ME, metabolizable energy; EE, ether extract; GE, gross energy.

1) The unit of chemical composition was expressed as % (dry matter basis), and the unit of energy values (GE, DE, ME, and NE) was expressed as MJ/kg dry matter.

2) Eight rapeseed meal samples included 5 samples used in the current study and 3 samples reported by Li et al [29] and Liu et al [6]. All experiments were conducted using same chambers and procedures.
