# Supplementary material for: Prediction of net energy values in expeller-pressed and solvent-extracted rapeseed meal for growing pigs
Source: Anim Biosci. 2020 Apr 12;34(1):109–18. doi: 10.5713/ajas.19.0962 (PMC7888491; doi:10.5713/ajas.19.0962)
Supplement: Supplementary file 1 [file ajas-19-0962-suppl.pdf]

**Supplementary Table S1.** Analyzed nutrient composition of 12 samples of rapeseed meal collected in the experiment (%, DM basis)[1)]

| Item[2)] | EP-RSM[3)] | | | | | | SE-RSM[3)] | | | | | |
|---|---|---|---|---|---|---|---|---|---|---|---|---|
| | 1 | 2 | 3 | 4 | 5 | 6 | 7 | 8 | 9 | 10 | 11 | 12 |
| DM, % | 92.20 | 91.92 | 93.53 | 91.65 | 92.58 | 92.55 | 89.20 | 90.60 | 89.71 | 91.48 | 89.90 | 90.82 |
| GE, MJ/kg | 21.26 | 20.94 | 21.35 | 20.56 | 20.85 | 21.06 | 19.47 | 19.37 | 19.59 | 19.47 | 19.60 | 19.37 |
| CP, % | 43.72 | 39.76 | 37.70 | 39.75 | 39.41 | 42.35 | 41.50 | 39.44 | 41.82 | 39.92 | 41.03 | 41.83 |
| Ether extract, % | 7.32 | 8.40 | 11.27 | 6.55 | 9.10 | 8.08 | 0.87 | 1.67 | 0.97 | 2.58 | 1.71 | 1.68 |
| NDF, % | 51.15 | 32.85 | 41.00 | 32.34 | 41.45 | 41.54 | 33.11 | 32.70 | 34.26 | 37.27 | 30.84 | 39.83 |
| ADF, % | 25.49 | 21.66 | 24.18 | 21.75 | 21.19 | 21.26 | 20.82 | 19.93 | 21.55 | 21.50 | 20.04 | 24.79 |
| Crude fiber, % | 21.26 | 16.74 | 19.18 | 15.04 | 17.20 | 14.87 | 14.28 | 13.27 | 14.61 | 13.58 | 14.23 | 17.86 |
| Ash, % | 6.68 | 6.67 | 7.49 | 6.94 | 7.39 | 6.69 | 7.09 | 8.37 | 6.82 | 8.24 | 7.22 | 9.14 |

[1)]All samples were analyzed in duplicate.

[2)]DM, dry matter; GE, gross energy; CP, crude protein, NDF, neutral dietary fiber; ADF, acid dietary fiber.

[3)]EP-RSM, expeller-pressed rapeseed meal; SE-RSM, solvent-extracted rapeseed meal; number 3 and 4 of EP-RSM and number 10, 11, and 12 of SE-RSM were chosen to measure the NE in the current experiment.

**Supplementary Table S2.** Analyzed nutrient composition of 3 samples of rapeseed meal reported by Liu et al (2015) and Li et al (2017) (DM basis)[1)]

| Item | EP-RSM | SE-RSM | |
|---|---|---|---|
| | | 1 | 2 |
| Gross energy, MJ/kg | 21.33 | 19.54 | 19.66 |
| Crude protein, % | 39.2 | 42.4 | 39.67 |
| Starch, % | 3.7 | 2.8 | 6.5 |
| Ether extract, % | 9.5 | 1.1 | 1.9 |
| Neutral dietary fiber, % | 37.0 | 30.8 | 26.7 |
| Acid dietary fiber, % | 22.8 | 20.7 | 17.2 |
| Ash, % | 6.4 | 7.0 | 6.9 |
| DE, MJ/kg | 16.55 | 12.82 | 13.22 |
| ME, MJ/kg | 15.71 | 11.61 | 11.64 |
| NE, MJ/kg | 11.71 | 8.83 | 8.38 |

[1)]The EP-RSM and SE-RSM 1 were reported by Li et al (2017). The SE-RSM 2 was reported by Liu et al (2015). EP-RSM, expeller-pressed rapeseed meal; SE-RSM, solvent-extracted rapeseed meal; DE, digestible energy; DM, dry matter; ME, metabolizable energy; NE, net energy.
